# Preoperative Pericapsular Nerve Group Block Results in Less Pain, Decreased Narcotic Use, and Quicker Discharge Time Than No Block in Patients Who Were Surgically Treated for Femoroacetabular Impingement Syndrome

**DOI:** 10.1016/j.asmr.2022.06.004

**Published:** 2022-08-02

**Authors:** Robert Kollmorgen, Maleehah Umerani, James Gollon, Derek Fleming, Brian Lewis, Joshua Harris, Thomas Ellis

**Affiliations:** a Department of Orthopaedic Surgery, University of California San Francisco, Fresno, Hip Preservation, Fresno, California; b Hip Preservation, Upper Arlington, Ohio; c Orthopaedic One, Upper Arlington, Ohio; d Midwest Physician Anesthesia Services, Columbus, Ohio; e Department of Orthopaedic Surgery, Duke University Medical Center, Durham, North Carolina; f Houston Methodist Orthopedics & Sports Medicine, Outpatient Center, Houston, Texas, U.S.A.

## Abstract

**Purpose:**

To determine the effectiveness of pericapsular nerve group (PENG) block for patients surgically treated for femoroacetabular impingement syndrome (FAIS).

**Methods:**

Consecutive patients who underwent surgical treatment of FAIS either with or without preoperative PENG block by a single surgeon were retrospectively identified. Twenty-five patients who received PENG block were matched 1:1 by age, sex, body mass index, and procedure to 25 patients who received no block (NB). A retrospective review of the medical records of consecutive patients undergoing the PENG block was performed. Outcome measures of postanesthesia care unit visual analog scale initial (PACU VAS-initial), maximum (PACU VAS-max), discharge (PACU VAS-discharge), intraoperative fentanyl, pain medications in morphine equivalents (ME), and PACU to discharge times were recorded.

**Results:**

Twenty-five patients undergoing a PENG block and 25 patients who did not undergo a block (NB) were identified. No significant differences observed between age, sex, body mass index, surgery time, or procedures performed between the PENG and NB groups, *P* > .05. Significantly less VAS-initial was observed in the PENG group 3.7 ± 3.2, versus 5.5 ± 2.9 in the NB group, *P* = .04. Fentanyl usage intraoperatively was 137.3 ± 53.3 μg versus 108.5 ± 39.6 μg in NB versus PENG group respectively, *P* = .04. Narcotic use was 50.29 ± 11.2 ME versus 34.3 ± 12.1 ME in NB versus PENG group respectively, *P* = .001. PACU to discharge time was 95.8 ± 31 minutes versus 81.5 ± 19 minutes in NB versus PENG group, respectively, *P* = .05. No patient in the PENG group demonstrated a motor nerve palsy.

**Conclusions:**

For patients undergoing hip arthroscopy for FAIS, the addition of a preoperative PENG block showed a significant decrease in initial PACU pain, PACU narcotic consumption, intraoperative fentanyl usage, and quicker time to discharge without complications when compared to a no block, post-free control group.

**Level of Evidence:**

III, retrospective cohort study.

The utility of hip arthroscopy (HA) in the United States has rapidly grown over the last 15 years to more than 53,103 cases performed from 2010 to 2017.^1^ HA is commonly used to treat labral tears, femoroacetabular impingement syndrome (FAIS), and intra-articular or peritrochanteric conditions.^2^, ^3^, ^4^ Anesthesia for HA has varied from general to various regional anesthetic options. Regional anesthesia techniques have shown to be safe and consistently has shown to provide decreased postoperative pain and analgesic use.^5^ Current options for peripheral nerve blocks vary from spinal, femoral, iliolumbar, fascia iliac, and recently the pericapsular nerve group (PENG) block. The optimal method has yet to be determined.

The use of a PENG block has previously been described in patients with hip fractures, arthritis, and recently HA.^6^, ^7^, ^8^, ^9^ The PENG block is performed under an ultrasound-guided technique allowing local anesthetic to be injected into the superior pubic ramus near the iliopectineal bursa^9^, ^10^, ^11^ (Fig 1). The block targets the branches of the femoral and obturator nerve. Additional local anesthetic can be infiltrated to affect the genitofemoral and lateral femoral cutaneous nerves.^6^^,^^9^

**Fig 1:**
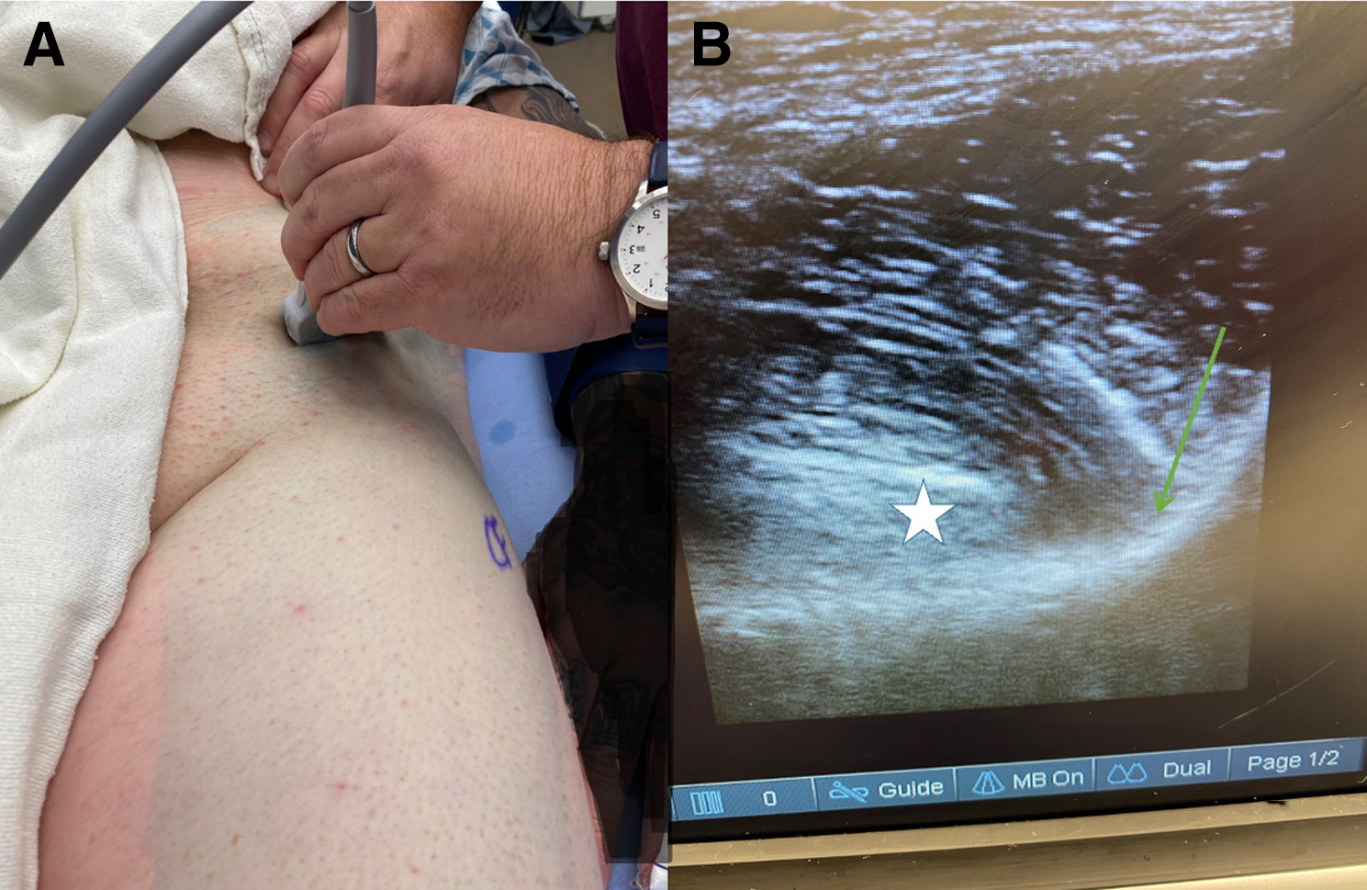
(A) Patient is in the supine position undergoing left PENG block. The ultrasound probe is over the anterior superior iliac spine and turned 45° to be in line with the iliopectineal eminence (IPE). (B) Needle is inserted in plane between the IPE and psoas tendon. Green arrow shows the IPE; star shows the psoas tendon. (PENG, pericapsular nerve group.)

The optimal choice for regional anesthesia for HA remains a subject of debate. The purpose of this study is to determine the effectiveness of the PENG block for patients treated for FAIS. We hypothesized that a consecutive series of patients undergoing HA receiving the PENG block would experience less pain, demonstrate decreased narcotic use, and show a quicker time to discharge as compared to a no-block (NB) control group.

## Methods

After institutional review board approval, 50 consecutive patients who underwent surgical treatment of FAIS either with or without preoperative PENG block by a single surgeon (T.E.) were retrospectively identified. Inclusion criteria was any patient who underwent primary HA for FAIS. Patients were excluded if they were undergoing revision HA and/or HA with concurrent periacetabular osteotomy or femoral osteotomy. Twenty-five patients who received PENG block were matched 1:1 by age, sex, body mass index, and procedure to 25 patients who received NB.

PENG blocks were performed by multiple anesthesiologists using a standard ultrasound-guided technique in the preoperative holding area.^11^ In brief, the block was performed with the patient in the supine position (Fig 1). The ultrasound probe was used and placed over the anterior inferior iliac spine and rotated 45° to be in position with the pubic ramus. The needle was inserted using an in-plane method to ensure needle placement was between the pubic ramus and psoas tendon.^10^^,^^11^ With a 22-gauge (millimeter) needle, 20 mL of ropivacaine was injected and later combined with a general anesthetic. The NB group received only general anesthesia, and neither group received intraoperative local anesthetic.

Surgeries were performed using the supine position with a Smith & Nephew distraction table (Watford, UK) with a previously described post-free technique.^12^ To summarize, the patient was placed in the modified supine position using the Pink Pad (Xodus Medical, Kensington, PA). Standard anterolateral, mid-anterolateral, and distal anterolateral portals were used. Acetabuloplasty, femoroplasty, and/or labral repair were performed as indicated. The capsule was meticulously closed in all patients.

Outcome measures were extracted from chart review: Outcome measures of postanesthesia care unit visual analog scale initial (PACU VAS-initial), maximum (PACU VAS-max), discharge (PACU VAS-discharge), intraoperative fentanyl use, pain medications in morphine equivalents (ME), and PACU to discharge times were recorded. VAS has been validated to measure pain levels and has been previously validated for HA to measure chronic and acute pain.^13^ VAS was documented by the nursing staff in the PACU on a Likert scale, 0 to 10 and it is based on the question “How much pain do you have in your hip?” A measurement of 0 was no pain while a measurement of 10 was the worst pain possible. Three measurements of VAS were extracted from the chart: VAS initial (the first score that was reported upon entering the PACU), VAS maximum (maximum documented PACU score), and the VAS at discharge. To have standardization of reported opioid usage, all opioid narcotics administered were converted to MEs, in milligrams.^14^

Microsoft Excel (Microsoft, Redmond, WA) was used to perform statistical analysis. The Welch *t* test was used to compare the noncategorical data with a significance set at *P* ≤ .05 and the Fisher exact test was used for categorical data with significance set at *P* ≤ .05. An a priori power analysis was performed and affirmed 17 patients would be required in each group to observe a *P* < .05 significance, 1– β = 0.8. Based on previous studies to observe a 2-point difference in the VAS, 25 patients were selected.^15^

## Results

Fifty consecutive patients met the inclusion criteria. Procedures included femoral neck osteochondroplasty, acetabuloplasty, labral repair, and capsular closure (Table 1). The PENG block group consisted of 13 female and 12 male patients and the NB group consisted of 14 female and 11 male patients. No significant differences were observed between age, sex, body mass index, surgery time, or procedures performed, *P* > .05 (Tables 1 and 2). Femoral neck osteoplasty was performed on 22 patients in the NB group and 24 patients in the PENG block group, *P* = .94. Labral repair was performed on 19 patients in the NB group and 23 patients in the PENG block group and *P* = .97 (Table 1). VAS initial was 5.5 ± 2.9 versus 3.7 ± 3.2 in the NB versus PENG groups respectively, *P* = .04. Significant differences were not observed between VAS max nor VAS at discharge, *P* > .05. Fentanyl usage intraoperatively was 137.3 ± 53.3 μg versus 108.5 ± 39.6 μg in the NB versus PENG group respectively, *P* = .05 (Table 3). Total pain medications required were 50.29 ± 11.2 ME versus 34.3 ± 12.1 ME in the NB versus PENG group, respectively, *P* = .001 (Table 3). The time to discharge was 95.8 ± 31 minutes versus 81.5 ± 19 minutes in the NB versus PENG group, respectively *P* = .01 (Table 3). No complications were reported in either group, and no patient in the PENG group demonstrated motor nerve palsy.

**Table 1:** Procedures Performed for the Study Groups

CPT Code	Procedure	PENG N (%)	No Block N (%)	*P* Value
29914	Femoral neck osteoplasty	24 (96)	22 (88)	.94
29915	Acetabuloplasty	1(4)	3 (12)	.94
29916	Labral repair	23 (92)	19 (76)	.97
29999-1	Capsular closure	25 (100)	25 (100)	1.00

NOTE. N represents the number of patients in each group, % the percentage of patients who had each procedure. *P* value, Fisher exact test, significance *P* ≤ .05.

CPT, Common Procedural Terminology; PENG, pericapsular nerve group.

**Table 2:** No Block Versus PENG Block Demographics

	No Block (Range)	±SD	95% CI	PENG (Range)	±SD	95% CI	*P* Value
Age, y (range)	25.52 (15-44)	8.8	21.8-29.2	26.5 (14-48)	10.4	22.2-30.8	.71
Sex, N (% female)	14 (56%)			13 (52%)			.84
BMI	26.44 (17.7-36.6)	4.4	24.6-28.3	26 (18-36)	5.1	23.9-28.2	.77

NOTE. Age represented in years, and sex is represented in number and percentage of females. *P* ≤ .05.

BMI, body mass index; CI, 95% confidence interval; PENG, pericapsular nerve group; SD, standard deviation.

**Table 3:** Outcome Measures

	No Block (Range)	±SD	95% CI	PENG (Range)	±SD	95% CI	*P* Value
Surgery time, min	103.76 (65-143)	20.5	95.3-112.2	106 (56-260)	36.2	91-121	.78
VAS initial	5.5 (0-10)	2.9	4.3-6.7	3.7 (0-10)	3.2	2.3-5	**.04**∗
VAS max	7.4 (3-10)	1.6	6.8-8.1	6.5 (2-10)	2	5.7-7.4	.08
VAS discharge	5.4 (1-9)	2.1	4.5-6.2	4.5 (0-7)	1.5	3.8-51	.1
Intraoperative fentanyl, μg	137.2 (80-250)	53.3	115.2-159.2	108.5 (50-200)	39.6	92-125	**.04**
Pain medications, ME	50.29 (26.2-79)	11.2	45.7-55	34.3 (14-59.2)	12.1	29.3-39	**.001**
PACU to discharge, min	95.8 (60-218)	31	83-109	81.5 (47-121)	19	73.7-89.4	**.05**

NOTE. Surgery time and PACU to discharge times represented in minutes. VAS in PACU initial, VAS maximum, max, are rated on a 0-10 scale. Micrograms and PACU Pain Meds are represented in ME.

CI, 95% confidence interval; ME, morphine equivalents; PACU, postanesthesia care unit; PENG, pericapsular nerve group; SD, standard deviation; VAS, visual analog scale.

∗ Bolded *P* values indicate significant scores, *P* ≤ .05.

## Discussion

The most important finding of this study is that the addition of a PENG block resulted in significantly less initial PACU pain. In addition, the PENG block has shown to significantly decrease intraoperative and total narcotic requirements. Lastly, the PENG block has shown to significantly decrease time to discharge by 14 minutes.

With the rise in popularity of HA, hip preservationists are searching for most optimal techniques to control pain and decrease discharge times for outpatient surgeries. Peripheral nerve blocks, in addition to general anesthesia, have shown to effectively manage pain and decrease the use of narcotics.^16^^,^^17^ A recent report showed a preoperative lumbar plexus block in addition to general anesthesia helped decrease the postoperative pain for HA patients as compared with fascia iliaca block and straight general anesthesia.^18^ A randomized control trial by Xing et al.^19^ observed that a femoral nerve block can help improve early pain control. However, pain scores between the femoral nerve block and control group showed no difference 1 to 7 days postoperatively. While femoral nerve and fascia iliaca blocks have shown positive results for postsurgery pain relief, the obturator and accessory obturator nerves need to be addressed for effective pain relief.^16^^,^^20^^,^^21^ According to a study by Blackwell et al.,^22^ a quadratus lumborum block is more effective for HA than a femoral nerve and fascia iliaca block as the quadratus lumborum block led to reduced narcotic usage, lower pain scores at the time of discharge, and a shorter PACU time.

Regional anesthesia has shown favorable outcomes in open and arthroscopic procedures. However, the optimal method for peripheral nerve block for HA has yet to be discerned.^23^, ^24^, ^25^ A systematic review regarding the use of regional blocks with HA was performed by Kay et al.^26^ The blocks in this study included femoral, lumbar plexus, fascia iliaca, and L1 and L2 paravertebral nerve blocks.^26^ The authors concluded the use of peripheral nerve blocks led to a decrease in opioid usage and lower postoperative pain. Limiting the use of fascia iliaca or femoral nerve blocks is recommended as the blocks may produce iatrogenic quadriceps weakness, and thus increase the risk of postoperative falls.^27^

Publications pertaining to the PENG block are limited to descriptive studies on the technique, but have limited investigational depth.^10^ Recently Girón-Arango et al.^9^ published a description of the PENG block for HA, but outcome measures were not reported. In addition, Talawar et al.^7^ reported on a PENG block with a lateral femoral cutaneous nerve block in a patient refusing a spinal anesthetic undergoing HA. Outcome reporting was limited in that the only outcome reported was that the anesthesia from the block lasted four and a half hours. Fernicola et al.^11^ recently described their technique of the PENG block for patient undergoing HA. This study was limited from the lack of patient outcomes. Orozco et al.^20^ performed the PENG block on 5 patients undergoing HA for FAIS and reported on initial postoperative as well as 48-hour postoperative VAS outcomes. Their results showed PACU VAS max to be 3 of 10, and at 48 hours reported a low VAS after HA.^20^ This study was limited by sample size. Our study demonstrated that in a matched cohort, the addition of a PENG block reduced intraoperative fentanyl use, decreased PACU narcotic consumption, and led to a quicker time to discharge. Future prospective studies including the PENG block may be of benefit to determine the optimal choice for regional anesthesia for HA.

### Limitations

Our study is not without limitations. This was a retrospective single-surgeon study, and the sample size, although adequate, is small. Since the PENG block was not compared with other regional methods, superiority cannot be demonstrated. Another limitation is the use of the VAS that is used for pain scoring in the PACU. These patient-reported outcomes were not standardized because the nursing staff would ask the patients at random points of time and pain ratings are subjective in nature. The PENG blocks were performed by multiple anesthesiologists and may expose our study to bias.

## Conclusions

For patients undergoing HA for FAIS, the addition of a preoperative PENG block showed a significant decrease in initial PACU pain, PACU narcotic consumption, intraoperative fentanyl usage, and quicker time to discharge without complications when compared with a no-block, post-free control group.

## Supplementary Material

ICMJE author disclosure forms
